# Major complications associated with unfavorable outcome in right‐sided large hemisphere infarctions: A single‐center study

**DOI:** 10.1002/brb3.3095

**Published:** 2023-06-07

**Authors:** Jie Li, Ping Zhang, Hong Chen, Yingying Liu, Xingrong Luo, Ju Zhou, Chun Wang

**Affiliations:** ^1^ Department of Neurology People's Hospital of Deyang City Deyang P. R. China

**Keywords:** complication, large hemispheric infarction, outcome, right hemisphere

## Abstract

**Objectives:**

To identify the major complications independently associated with unfavorable outcomes in right‐sided large hemisphere infarction (RLHI) patients.

**Methods:**

We retrospectively enrolled consecutive patients admitted within 24 h with the diagnosis of RLHI. The unfavorable outcome was defined as a modified Rankin Scale score of 4–6 at 3 months. Univariate and multivariate analyses were performed to identify the major complications independently associated with 3‐month unfavorable outcomes.

**Results:**

Of the 171 cases with RLHI included, 126 (73.7%) had unfavorable outcomes at 3 months: A total of 64 (37.4%) cases died, and 62 (36.3%) lived with severe disability. Stroke‐related complications occurred in 168 (98.2%) patients during hospitalization. The five most common stroke‐related complications were pulmonary infection (75.4%), electrolyte disorder (61.4%), hypoalbuminemia (49.1%), malignant brain edema (MBE) (48.5%), and hemorrhagic transformation (48.0%). RLHI patients with unfavorable outcomes had more frequent MBE (58.7% vs. 21.4%, *p* < .001), pulmonary infection (86.5% vs. 42.9%, *p* < .001), gastrointestinal bleeding (46.8% vs. 28.6%, *p* = .038), electrolyte disorder (68.3% vs. 40.5%, *p* = .001), acute renal failure (32.5% vs. 4.8%, *p* < .001), and hypoalbuminemia (61.1% vs. 11.9%, *p* < .001) than patients with favorable outcome. Multivariate analyses suggested that only MBE (adjusted OR 4.06, 95% confidence interval [CI] 1.14–14.48, *p* = .031), pulmonary infection (adjusted OR 4.69, 95%CI 1.48–14.85, *p* = .009), and hypoalbuminemia (adjusted OR 6.58, 95%CI 1.74–24.86, *p* = .005) were independently associated with 3‐month unfavorable outcome in patients with RLHI.

**Conclusions:**

Most of the RLHI patients have at least one stroke‐related complication during hospitalization, and nearly three‐quarters suffered unfavorable outcomes. Only MBE, pulmonary infection, and hypoalbuminemia are independently associated with 3‐month unfavorable outcome.

## Graphical abstract

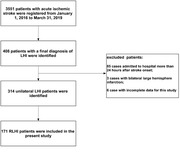

## 1 INTRODUCTION

Large hemispheric infarction is one of the most devastating conditions with high mortality and disability rate among acute ischemic stroke (AIS) patients (Huttner & Schwab, 2009; Uhl et al., 2004). The laterality of the affected hemisphere is an important determinant of the natural history of large hemisphere infarction (LHI) (Rastogi et al., 2015). The right hemisphere is often considered nondominant hemisphere, so that right hemisphere stroke may be less clinically evident, and the stroke severities assessed by the National Institutes of Health Stroke Scale (NIHSS) score are also weighted toward left hemisphere lesions (Fink et al., 2002). As a result, right hemisphere stroke can be deemed to be less severe than left‐sided, such that physicians and surgeons might be less aggressive in treatment (Fink et al., 2002).

Although it is reasonable to suspect that right hemisphere stroke can drive more favorable outcomes, strokes in the right hemisphere can cause more autonomic and cardiovascular dysfunction that affect mortality than left hemisphere stroke (Krause et al., 2017; Naver et al., 1996; Tokgözoglu et al., 1999; Yoon et al., 1997). Additionally, right hemisphere strokes might lead to lower rehabilitation potential due to visuospatial neglect or emotional indifference (Aszalós et al., 2002; Ween et al., 1996). We recently reported more than two thirds of right‐sided LHIs (RLHIs) suffered unfavorable outcomes and suggested that stroke lateralization was not an independent predictor of death and unfavorable outcomes in patients with LHIs (Li et al., 2021), which was in line with previous published systematic review and meta‐analysis (Almekhlafi et al., 2019; Rastogi et al., 2015). Despite this evidence, right hemisphere stroke has usually been considered to be less severe and has a higher risk of being excluded from clinical trials or established treatments (Fink et al., 2002).

It has been demonstrated that stroke‐related complications have a great influence on the death and unfavorable outcomes of AIS (Johnston et al., 1998; Weimar et al., 2002). Meanwhile, neurological impairment level is the most substantial factor in predicting the rate of complications (Roth et al., 2001). It is reasonable to suspect that stroke‐related complications might less frequently occur in right hemisphere stroke than in left hemisphere stroke. However, our previous study suggested that RLHI was independently associated with an increased risk of malignant brain edema (MBE) and a composite of cardiovascular events during hospitalization (Li et al., 2021). Several studies have also reported a higher incidence of pneumonia (Kemmling et al., 2013), and a higher risk of hemorrhagic transformation following intravenous thrombolysis in right hemisphere stroke patients (Audebert et al., 2011). Physicians need to be aware of the incidence of common stroke‐related complications of RLHI and their importance to patient outcome. Although our previous study has assessed the hemispheric differences in clinical characteristics, the incidence rate of stroke‐related complications, and outcomes of large hemisphere infraction patients, until recently, which complication might play a significant role in the poor outcomes of RLHI patients remains unclear.

Therefore, we conducted a retrospective cohort study using the prospectively acquired data of the Deyang stroke registry, to identify the major acute complications independently associated with 3‐month unfavorable outcomes in RLHI patients.

## 2 METHODS

### 2.1 Study design and subjects

AIS patients who were admitted to the People's Hospital of Deyang City within 24 h from symptoms onset were prospectively and consecutively registered from January 1, 2016 to March 31, 2019. We enrolled RLHI patients with computed tomography (CT) and/or magnetic resonance imaging (MRI) evidence of supratentorial cerebral infarction involving more than 50% of the right middle cerebral artery (MCA) region within 7 days after stroke onset, no matter the involvement of the right anterior cerebral artery (ACA) or posterior cerebral artery (PCA) (Uhl et al., 2004). Bilateral hemispheric strokes were excluded. All enrolled patients had a noncontract CT (NCCT) before initial treatment. A routine follow‐up NCCT or MRI was performed during the first 7 days after stroke onset. Other CT scans were performed when suffering neurological deterioration, to identify brain edema or hemorrhagic transformation. We excluded cases with incomplete hospital records or missing imaging that would prevent complete data collection. We also excluded cases with a preexisting score of more than 2 on the modified Rankin scale (mRS) and lived dependently (de Haan et al., 1995). The mRS score is a scale of 0–6, which categorized the magnitude of neurologic disability into one of the seven exclusive categories (de Haan et al., 1995): 0 (no symptoms), 1 (no significant disability), 2 (slight disability), 3 (moderate disability), 4 (moderately severe disability), 5 (severe disability), 6 (death). The study was approved by the Ethics Committee of People's Hospital of Deyang City (Reference No. 2011‐04‐134). Written informed consent was obtained from all patients before they were enrolled, or from their legal representative if the patient lost the capacity to give informed consent.

### 2.2 Data collection and outcome

Baseline data on age, gender, admission delay, baseline NIHSS score, baseline systolic and diastolic blood pressure, serum glucose on admission, and vascular risk factors were collected, which has been described in our previous study (Li et al., 2018). The potential etiology of LHI was classified as cardio‐embolism or not according to the Trial of Org 10172 in Acute Stroke Treatment (TOAST) criteria (Adams et al., 1993). Two experienced neurologists who were blinded to clinical information independently evaluated the brain imaging. Disagreement was resolved through discussion or further consulting with a third neurologist. The hyperdense MCA sign (HDMCAS) and Alberta Stroke Program Early CT Score (ASPECTS) were assessed on the pretreatment NCCT (Pexman et al., 2001; Topcuoglu et al., 2014). The basal ganglia involvement was defined as the presence of infarction on CT and/or MRI imaging, involving caudate, lentiform nucleus (putamen and globus pallidus), or both. Final infarct territory on the following‐up imaging within 7 days after stroke onset was dichotomized into the right MCA territory, the right MCA and ACA territory (MCA + ACA), and the right MCA and PCA territory (MCA + PCA). Hemorrhagic transformation during hospitalization was classified as hemorrhagic infarct and parenchymal hematoma based on follow‐up CT or MRI according to the European Cooperative Acute Stroke Study (ECASS) II criteria (Hacke et al., 1998). MBE was defined as the development of clinical signs of herniation (including decrease in consciousness and/or anisocoria), accompanied by a midline shift of ≥5 mm at the septum pellucidum or pineal gland with effacement of the basal cisterns on follow‐up imaging (Kimberly et al., 2018), excluding patients with parenchymal hematoma (defined as hemorrhage of brain parenchyma with mass effect according to the ECASS II criteria (Hacke et al., 1998)).

In‐hospital treatments (defined as acute treatment administered during hospitalization) analyzed in our study included thrombolysis, endovascular interventions, decompressive hemicraniectomy (DHC), mechanical ventilation, osmotic agents (such as mannitol), antiplatelet agents, and statins. Thrombolysis or endovascular interventions were performed according to the Chinese guidelines, which had the similar inclusion and exclusion criteria compared with the American guideline (Jauch et al., 2013; Powers et al., 2015). The final treatment decision was made in consultation with the neurologists, patients, and their families. DHC was considered for RLHI patients with significant neurological deterioration and MBE, and the final decision was made in consultation with neurosurgeons and the patient's family. Stroke‐related complications, including neurological and medical complications during hospitalization, were reviewed by data collectors who were not involved in the present study from hospital records when the patient was discharged (Li et al., 2019). Neurological complications included MBE, hemorrhagic transformation, poststroke seizures/epilepsy, and early recurrent stroke, whereas medical complications included composite of cardiovascular events, pulmonary infection, urinary tract infection, gastrointestinal bleeding, electrolyte disorder, acute renal failure, hypoalbuminemia, urinary incontinence, deep venous thrombosis, and bedsore, which have been elaborated in our previous study (Li et al., 2019, 2021). Composite of cardiovascular events in our study was defined as a composite of myocardial infarction, acute heart failure, or any sudden cardiac death (Park et al., 2020). Hypoalbuminemia in the present study was defined as serum albumin level <35 g/L during hospitalization (Dziedzic et al., 2006).

Independent outcome assessments were performed by researchers who were blind to the clinical information, at 3 months after stroke by telephone interview or by mail. The primary outcome measures in our study were 3‐month unfavorable outcome (defined as an mRS score of 4–6, including death or living with a severe disability (de Haan et al., 1995)).

### 2.3 Statistical analyses

Baseline characteristics, in‐hospital treatment, and stroke‐related complications were compared between RLHI patients with 3‐month unfavorable and favorable outcomes. The *χ* (Huttner & Schwab, 2009) tests or Fisher's exact tests were used for intergroup differences in categorical variables, whereas Student's *t*‐tests or the Mann–Whitney *U* test were used for intergroup differences in continuous variables. Univariate analysis was performed to test variables that might affect the outcomes. The included variables were (1) age, (2) baseline NIHSS score, (3) vascular risk factors, (4) imaging characteristics, (5) in‐hospital treatment, and (6) stroke‐related complications. Multivariate analysis was performed by using the forced entry method, including variables with *p* < .1 in univariate analyses, to identify the factors independently associated with 3‐month unfavorable outcome in RLHI patients (excluding stroke‐related complications). Then, we identified the major complications via adjusting for confounders, which had a significant association with 3‐month unfavorable outcome in multivariate analysis. The 95% confidence intervals (CIs) were calculated to describe the precision of the estimates. All statistical analyses were performed using SPSS v21.0 (IBM, Chicago, IL, USA). Two‐sided *p* value <.05 was considered to be statistically significant.

## 3 RESULTS

**FIGURE 1:**
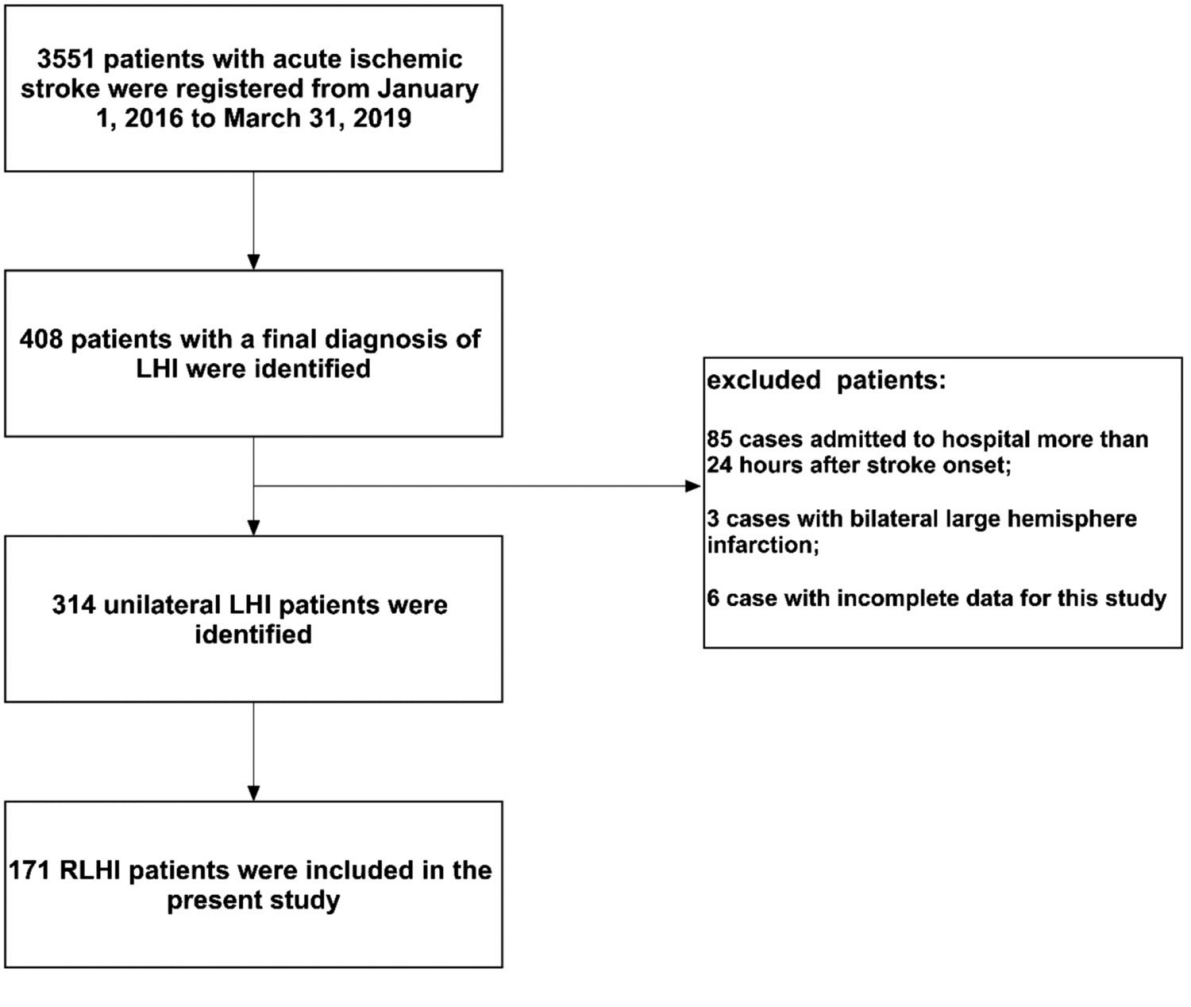
Flow diagram of included and excluded patients.

During the study period, 3551 AIS patients were consecutively registered. Of those patients, 171 (4.8%) RLHI patients admitted within 24 h were enrolled in the present study (mean age: 69.1 ± 14.2 years; 91 [53.2%] male; median NIHSS score on admission: 18). The median time for the diagnosis of LHI after stroke was 24 h (interquartile range [IQR]: 5–48 h). A flow diagram of included and excluded patients in our study is provided in Figure 1. All RLHI patients received at least 1 CT scan, and 76 (44.4%) cases received MRI. During hospitalization, 34 (19.9%) cases were treated with thrombolysis, 21 (12.3%) were treated with endovascular interventions, 24 (14.0%) cases received DHC, and 61 (35.7%) received mechanical ventilation. The median length of hospital stay was 14 days (IQR: 7–20 days). Among the entire cohort, 126 (73.7%) patients had unfavorable outcomes at 3 months: A total of 64 (37.4%) cases died, and 62 (36.3%) lived with a severe disability, whereas 42 (24.6%) cases had favorable outcomes. The remaining 3 (1.7%) cases were lost to follow‐up at 90 days. Baseline characteristics, in‐hospital treatment, and clinical outcomes of patients with RLHI have been described in detail in our previous published study (Li et al., 2021).

### 3.1 Baseline characteristics and in‐hospital treatment between outcome groups

Baseline characteristics and in‐hospital treatment were compared between RLHI patients with or without unfavorable outcomes (Table 1). Patients with unfavorable outcomes were older (72.1 ± 13.2 vs. 59.8 ± 13.2, *p* < .001) and had higher systolic blood pressure (143.2 ± 25.9 vs. 132.9 ± 18.2 mmHg, *p* = .006) and higher serum glucose (8.5 ± 2.5 vs. 7.3 ± 2.1 mmol/L, *p*  .004) on admission. The median baseline NIHSS score was 19 in patients with unfavorable outcomes and 15 in those with favorable outcomes (*p* < .001). RLHI patients with unfavorable outcomes had more history of hypertension (65.1% vs. 26.2%, *p* < .001), diabetes mellitus (27.0% vs. 11.9%, *p* = .045), coronary heart disease (20.6% vs. 7.1%, *p* = .045), atrial fibrillation (73.8% vs. 57.1%, *p* = .042), and less history of current smoking (26.2% vs. 47.6%, *p* = .010).

**TABLE 1:** Baseline characteristics and in‐hospital treatment of right‐sided large hemisphere infarction (RLHI) patients with 3‐month unfavorable and favorable outcomes.

	Unfavorable (mRS score 4–6) (*n* = 126)	Favorable (mRS score 0–3) (*n* = 42)	*p* Value
Age (years), mean ± SD	72.1 ± 13.2	59.8 ± 13.2	**<.001** ^a^
Male, *n* (%)	67 (53.2)	24 (57.1)	.655^b^
Time from onset (h), median (IQR)	5 (3–10)	5 (3–24)	.564^c^
NIHSS score on admission, median (IQR)	19 (16–23)	15 (10–18)	**<.001** ^c^
SBP on admission (mmHg)	143.2 ± 25.9	132.9 ± 18.2	**.006** ^a^
DBP on admission (mmHg)	84.0 ± 17.2	80.0 ± 12.9	.114^a^
Serum glucose on admission (mmol/L)	8.5 ± 2.5	7.3 ± 2.1	**.004** ^a^
Risk factors, *n* (%)			
Hypertension	82 (65.1)	11 (26.2)	**<.001** ^b^
Diabetes mellitus	34 (27.0)	5 (11.9)	**.045** ^b^
Dyslipidemia	26 (20.6)	9 (21.4)	.913^b^
Coronary heart disease	26 (20.6)	3 (7.1)	**.045** ^b^
Atrial fibrillation	93 (73.8)	24 (57.1)	**.042** ^b^
Rheumatic heart disease	17 (13.5)	8 (19.0)	.381^b^
Current smoking	33 (26.2)	20 (47.6)	**.010** ^b^
Previous ischemic stroke/TIA	22 (17.5)	5 (11.9)	.396^b^
Previous ICH	2 (1.6)	0 (0)	1.000^b^
Cardio‐embolism, *n* (%)	98 (77.8)	26 (61.9)	**.043** ^b^
HDMCAS, *n* (%)	37 (29.4)	14 (33.3)	.699^b^
ASPECTS, median (IQR)	6 (3–8)	6 (5–9)	**.033** ^c^
Basal ganglia involvement	90 (71.4)	29 (69.0)	.769^b^
Final infarct territory			**.019** ^b^
MCA	99 (78.6)	40 (95.2)	
MCA + PCA	1 (0.8)	0 (0)	
MCA + ACA	26 (20.6)	2 (4.8)	
In‐hospital treatments, *n* (%)			
Thrombolysis	25 (19.8)	9 (21.4)	.825^b^
Endovascular interventions	18 (14.3)	3 (7.1)	.225^b^
DHC	21 (16.7)	3 (7.1)	.127^b^
Mechanical ventilation	55 (43.7)	5 (11.9)	**<.001** ^b^
Osmotic agents	125 (99.2)	38 (90.5)	**.018** ^b^
Antiplatelets	81 (64.3)	34 (81.0)	**.044** ^b^
Statins in acute phase	66 (52.4)	32 (76.2)	**.007** ^b^

*Note*: *p* Values of <.1 are shown in bold.

Abbreviations: ACA, anterior cerebral artery; ASPECTS, Alberta Stroke Program Early Computed Tomography Score; DBP, diastolic blood pressure; DHC, decompressive hemicraniectomy; HDMCAS, the hyperdense middle cerebral artery sign; ICH, intracerebral hemorrhage; IQR, interquartile range; MCA, middle cerebral artery; mRS, modified Rankin scale; NIHSS, National Institutes of Health Stroke Scale; PCA, posterior cerebral artery; SBP, systolic blood pressure; SD, standardized difference; TIA, transient ischemic stroke.

^a^ Student *t*‐test.

^b^ *χ* ^2^ test.

^c^ Mann–Whitney *U* test.

There was no difference in the gender, median admission delay, diastolic blood pressure, and other vascular risk factors between the two groups (all *p* > .05). When stroke etiology of RLHI is concerned, patients with unfavorable outcome showed a higher proportion of cardio‐embolism (77.8% vs. 61.9%, *p* = .043) than the favorable outcome group. The unfavorable outcome group had a lower ASPECTS (IQR 3–8 vs. 5–9, *p* = .033) and a higher proportion of infarction involving the ACA territory (MCA + ACA) (20.6% vs. 4.8%, *p* = .019); however, the presence of HDMCAS and basal ganglia involvement were comparable between the two groups (both *p* > .05). For the in‐hospital treatments of RLHI, patients with unfavorable outcomes more frequently received mechanical ventilation (43.7% vs. 11.9%, *p* < .001) and osmotic agents (99.2% vs. 90.5%, *p* = .018), nevertheless, less frequently received antiplatelets (64.3% vs. 81.0%, *p* < .001) and statins (52.4% vs. 76.2%, *p* = .007) in the acute phase of stroke. There was no difference in the administration rates of thrombolysis, endovascular interventions, and DHC (all *p* > .05).

### 3.2 Stroke‐related complications in RLHI

**FIGURE 2:**
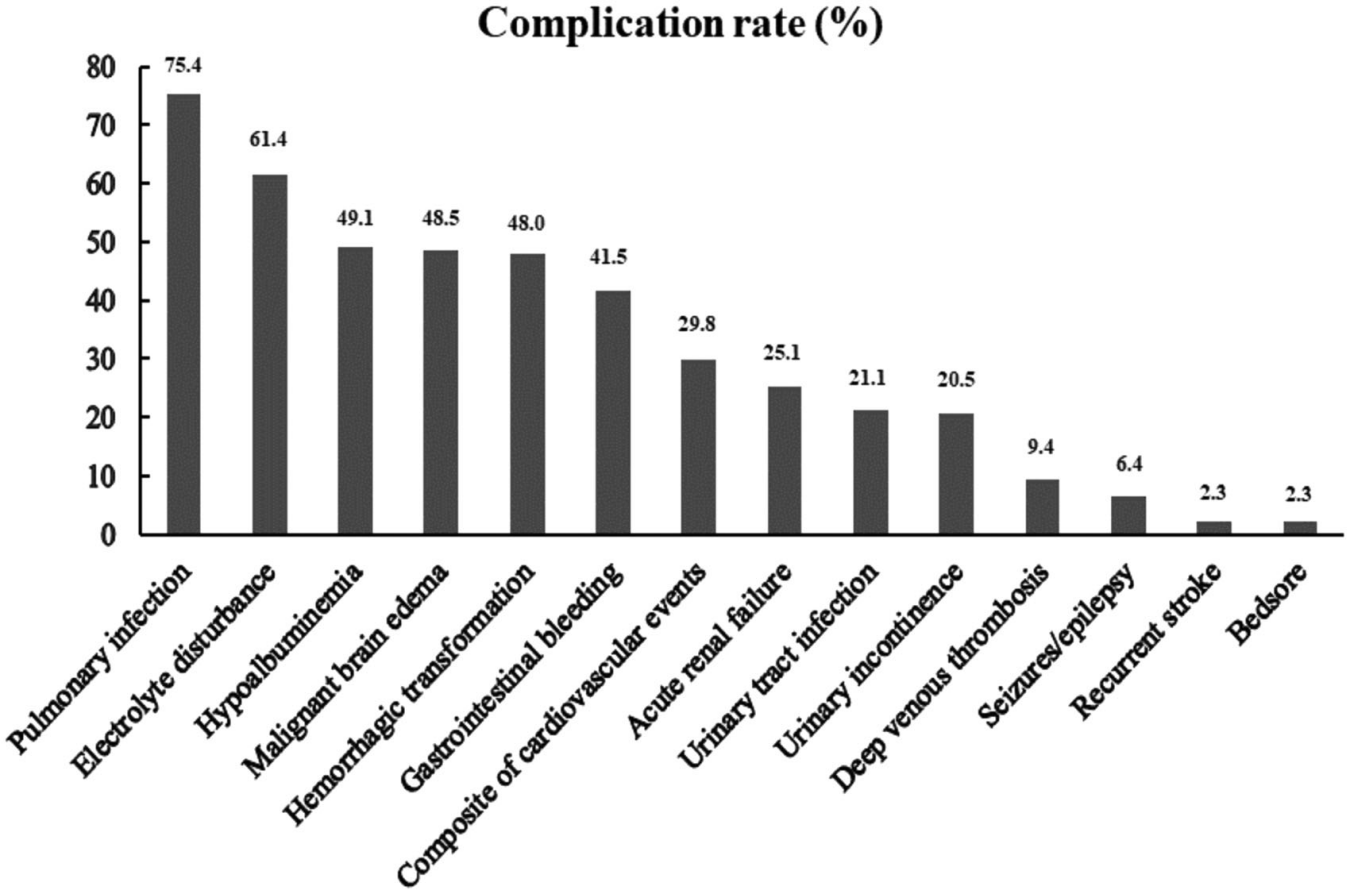
The incidence of stroke‐related complications in the entire cohort of right‐sided large hemisphere infarction (RLHI) patients.

Stroke‐related complications occurred in 168 (98.2%) patients during hospitalization. The incidence of complications was ranked from high to low as follows (Figure 2): pulmonary infection (75.4%), electrolyte disorder (61.4%), hypoalbuminemia (49.1%), MBE (48.5%), hemorrhagic transformation (48.0%), gastrointestinal bleeding (41.5%), composite of cardiovascular events (29.8%), acute renal failure (25.1%), urinary tract infection (21.1%), urinary incontinence (20.5%), deep venous thrombosis (9.4%), poststroke seizures/epilepsy (6.4%), early recurrent stroke (2.3%), and bedsore (2.3%).

RLHI patients with unfavorable outcomes had higher incidence rates of MBE (58.7% vs. 21.4%, *p* < .001), pulmonary infection (86.5% vs. 42.9%, *p* < .001), gastrointestinal bleeding (46.8% vs. 28.6%, *p* = .038), electrolyte disorder (68.3% vs. 40.5%, *p* = .001), acute renal failure (32.5% vs. 4.8%, *p* < .001), and hypoalbuminemia (61.1% vs. 11.9%, *p* < .001). Meanwhile, the unfavorable outcome group had a trend to suffer more composite of cardiovascular events during hospitalization (33.3% vs. 19.0%, *p* = .079). However, there was no significant difference in the events rates of hemorrhagic transformation (including hemorrhagic infarct and parenchymal hematoma), poststroke seizures/epilepsy, recurrent stroke, urinary tract infection, urinary incontinence, deep venous thrombosis, and bedsore between two groups (all *p* > .05) (Table 2).

**TABLE 2:** Stroke‐related complication during hospitalization of right‐sided large hemisphere infarction (RLHI) patients with 3‐month unfavorable and favorable outcomes.

	Unfavorable (mRS score 4–6) (*n* = 126)	Favorable (mRS score 0–3) (*n* = 42)	*p* Value
Stroke‐related complications, *n* (%)	125 (99.2)	40 (95.2)	.155^a^
Neurological complications, *n* (%)			
Malignant brain edema	74 (58.7)	9 (21.4)	**<.001**
Hemorrhagic transformation	63 (50.0)	18 (42.9)	.422
Hemorrhagic infarct	24 (19.0)	6 (14.3)	.485
Parenchymal hematoma	39 (31.0)	12 (28.6)	.771
Seizures/epilepsy	10 (7.9)	1 (2.4)	.368
Recurrent stroke	4 (3.2)	0 (0)	.573^a^
Medical complications, *n* (%)			
Composite of cardiovascular events	42 (33.3)	8 (19.0)	**.079**
Pulmonary infection	109 (86.5)	18 (42.9)	**<.001**
Urinary tract infection	27 (21.4)	8 (19.0)	.742
Gastrointestinal bleeding	59 (46.8)	12 (28.6)	**.038**
Electrolyte disorder	86 (68.3)	17 (40.5)	**.001**
Acute renal failure	41 (32.5)	2 (4.8)	**<.001**
Hypoalbuminemia	77 (61.1)	5 (11.9)	**<.001**
Urinary incontinence	28 (22.2)	7 (16.7)	.443
Deep venous thrombosis	13 (10.3)	3 (7.1)	.762
Bedsore	4 (3.2)	0 (0)	.573^a^

*Note*: *p* values of <.1 are shown in bold.

Abbreviation: mRS, modified Rankin scale.

^a^ Fisher exact test.

### 3.3 Factors associated with 3‐month unfavorable outcome in RLHI patients (excluding stroke‐related complications)

Variables that may have potential effects on the 3‐month unfavorable outcome in univariate analysis (*p* < .1) were included in multivariate logistic regression, and the results are shown in Table 3. After adjusting for potential confounders excluding stroke‐related complications, age (OR 1.12; 95%CI 1.05–1.19), baseline NIHSS score (OR 1.14; 95%CI 1.02–1.27), ASPECTS (OR 0.71; 95%CI 0.56–0.89), and mechanical ventilation (OR 8.40; 95%CI 1.80–39.28) were independently associated with 3‐month unfavorable outcome in patients with RLHI (all *p* < .05).

**TABLE 3:** Multivariate analysis for factors associated with 3‐month unfavorable outcome in right‐sided large hemisphere infarction (RLHI) patients (excluding stroke‐related complications).

Variables	Unfavorable outcomes OR (95%CI)	*p* Value
Age (years)	1.12 (1.05–1.19)	**<.001**
NIHSS score on admission	1.14 (1.02–1.27)	**.018**
SBP on admission (mmHg)	0.99 (0.97–1.02)	.440
Serum glucose on admission (mmol/L)	1.22 (0.90–1.64)	.197
Hypertension	2.62 (0.72–9.52)	.144
Diabetes mellitus	1.06 (0.25–4.58)	.936
Coronary heart disease	1.44 (0.20–10.56)	.723
Atrial fibrillation	0.57 (0.07–4.85)	.603
Current smoking	1.50 (0.46–4.85)	.500
Cardio‐embolism	1.63 (0.19–14.05)	.657
ASPECTS	0.71 (0.56–0.89)	**.003**
Final infarct territory		.714
MCA	Reference	–
MCA + PCA	NA	1.000
MCA + ACA	2.26 (0.32–15.89)	.412
Mechanical ventilation	8.40 (1.80–39.28)	**.007**
Osmotic agents	6.42 (0.21–194.67)	.285
Antiplatelets	0.63 (0.15–2.67)	.529
Statins in acute phase	0.57 (0.15–2.13)	.401

*Note*: Variables that had a potential association with 3‐month unfavorable outcome in univariate analysis (*p* < .1) were listed. *p* Values of <.05 are shown in bold.

Abbreviations: ACA, anterior cerebral artery; ASPECTS, Alberta Stroke Program Early Computed Tomography Score; CI, confidence interval; MCA, middle cerebral artery; NIHSS, National Institutes of Health Stroke Scale; PCA, posterior cerebral artery; SBP, systolic blood pressure.

### 3.4 Major complications associated with 3‐month unfavorable outcome in RLHI patients

After adjusting for age, baseline NIHSS score, ASPECTS, and mechanical ventilation, which had a significant association with 3‐month unfavorable outcome in multivariate analysis, only MBE (adjusted OR 4.06, 95%CI 1.14–14.48, *p* = .031), pulmonary infection (adjusted OR 4.69, 95%CI 1.48–14.85, *p* = .009), and hypoalbuminemia (adjusted OR 6.58, 95%CI 1.74–24.86, *p* = .005) were independently associated with 3‐month unfavorable outcome in patients with RLHI (Table 4).

**TABLE 4:** Multivariate analysis to identify the major complications associated with 3‐month unfavorable outcome in right‐sided large hemisphere infarction (RLHI) patients.

Variables	Unfavorable outcomes OR (95%CI)	*p* Value
**Neurological complications**		
Malignant brain edema	4.06 (1.14–14.48)	**.031**
Hemorrhagic transformation	1.72 (0.66–4.44)	.264
Seizures/epilepsy	9.46 (0.50–180.93)	.135
Recurrent stroke	NA	.999
**Medical complications**		
Composite of cardiovascular events	1.24 (0.38–4.01)	.719
Pulmonary infection	4.69 (1.48–14.85)	**.009**
Urinary tract infection	1.18 (0.39–3.53)	.770
Gastrointestinal bleeding	2.16 (0.76–6.10)	.149
Electrolyte disturbance	1.62 (0.60–4.38)	.346
Acute renal failure	3.67 (0.58–23.04)	.166
Hypoalbuminemia	6.58 (1.74–24.86)	**.005**
Urinary incontinence	0.42 (0.11–1.68)	.222
Deep venous thrombosis	1.49 (0.23–9.80)	.680
Bedsore	NA	.999

*Note*: Adjusted for age, baseline NIHSS score, baseline ASPECTS, and mechanical ventilation, which had a significant association with 3‐month unfavorable outcome in multivariate analysis. *p* Values of <.05 are shown in bold.

Abbreviations: ASPECTS, Alberta Stroke Program Early Computed Tomography Score; CI, confidence interval; NIHSS, National Institutes of Health Stroke Scale.

## 4 DISCUSSION

Although right hemisphere stroke has usually been considered to be less severe (Fink et al., 2002), our study suggested that 98.2% of the RLHI patients had at least one of the stroke‐related complications during hospitalization, and 73.7% of the RLHI patients suffered 3‐month unfavorable outcomes. The five most common stroke‐related complications in RLHI patients were pulmonary infection (75.4%), electrolyte disorder (61.4%), hypoalbuminemia (49.1%), MBE (48.5%), and hemorrhagic transformation (48.0%), which are in line with our previous published study conducted in LHI patients admitted within 30 days from onset (Li et al., 2019). Previous studies have suggested that about 24.2%–95% of AIS patients experienced one or more neurological and medical complications during hospitalization (Dromerick & Reding, 1994; Hong et al., 2008; Johnston et al., 1998; Roth et al., 2001). The incidence rate of stroke‐related complications in the present study was similar to those previous findings. It has been demonstrated that poststroke neurological complications mainly occur in the early course of neurological deterioration—within 48–72 h of stroke onset (Balami et al., 2011), whereas medical complications usually develop in the acute or subacute phase of stroke (within the first few weeks after stroke) (Kumar et al., 2010). In addition, the greater neurological deficit assessed by the NIHSS score is related to the higher incidence rate of stroke‐related complications (Roth et al., 2001). Different incidence rates of complications among different studies were most logically explained by the difference in the onset‐admission delay, stroke severity, and the definition and scope of the stroke‐related complications studied. The present study included RLHI patients admitted within 24 h from symptom onset, and we did not include patients with minor stroke, hemorrhagic stroke, and patients in stroke rehabilitation. Meanwhile, our study mainly focused on acute complications during hospitalization, we did not include subacute and chronic complications such as depression and cognitive impairment, because these complications are beyond the scope of the current study.

It is known that stroke‐related complications have a great influence on the death and functional outcome of AIS patients (Johnston et al., 1998; Weimar et al., 2002). Although it is common for RLHI patients to experience multiple medical and neurological complications (Audebert et al., 2011; Kemmling et al., 2013; Li et al., 2021), which complication might play a significant role in the poor outcome of RLHI patients remains unclear. In the present study, RLHI patients with unfavorable outcome had more frequent MBE, pulmonary infection, gastrointestinal bleeding, electrolyte disorder, acute renal failure, and hypoalbuminemia than patients with favorable outcome in univariate analysis. However, after adjusting potential confounders, only three major complications (MBE, pulmonary infection, and hypoalbuminemia) were independently associated with 3‐month unfavorable outcomes of RLHI patients. MBE is the most serious complication leading to early death and poor functional outcome in patients with LHIs (Liebeskind et al., 2019). Although DHC within 48 h has been proven to benefit LHI patients with MBE by randomized controlled trials (RCTs), only highly selected cases would be eligible for DHC according to guidelines (Rahme et al., 2012). Furthermore, because of its invasive nature and the need for multidisciplinary cooperation, DHC remains underutilized worldwide (Bar et al., 2011; Neugebauer et al., 2014). In our cohort, 48.5% of RLHI patients developed MBE, but only 14.0% underwent DHC, which was similar to the surgery rates reported in another real‐world observational study conducted in China (Hao et al., 2015). Even though RLHI patients have lower baseline NIHSS scores compared with left hemisphere strokes of equivalent volume, previous studies and systematic reviews have demonstrated that MBE appears to be more common in the right hemisphere infarction (Li et al., 2021; Rastogi et al., 2015). Further study is warranted to determine the early warning signs of MBE in patients with right hemisphere stroke, especially for those with lower NIHSS scores.

The most common complication in our cohort was pulmonary infection, with an incidence rate of 75.4%, which was similar to our previous study (Li et al., 2019). The incidence rate of pulmonary infection in our cohort is significantly higher than that of several studies conducted in stroke patients (Ji, Shen, et al., 2013; Smith et al., 2015; Teh et al., 2018; Yu et al., 2016). The higher incidence of pulmonary infection could be explained by immunodepression, impaired swallowing mechanism, impaired level of consciousness, and bedridden due to severe brain injury caused by RLHI (Yu et al., 2016). Several studies have suggested that pulmonary infection is closely associated with the prolonged length of hospital stay, increased medical cost, and poor functional outcomes in stroke patients (Hannawi et al., 2013; Teh et al., 2018; Yu et al., 2016). In the present study, after adjusting for age, stroke severity, and other confounders, the risk of 3‐month unfavorable outcome in RLHI patients complicated with pulmonary infection was 4.69 times higher than those without pulmonary infection, which was in line with previous studies (Hannawi et al., 2013; Teh et al., 2018; Yu et al., 2016). An observational study found that poststroke pneumonia is closely associated with the development of several non‐pneumonia complications, and pneumonia might be a risk marker for the development of several non‐pneumonia complications after stroke (Ji, Wang, et al., 2013). Because most pulmonary infection is potentially preventable or treatable, we should pay more attention to the prevention and control of pulmonary infection in RLHI patients, because of the higher incidence rate and concomitant unfavorable outcome.

The study found that the incidence rate of hypoproteinemia in RLHI patients was as high as 49.1%, which was similar to the results of previous studies (Dziedzic et al., 2007; Vahedi et al., 2011). Multivariate analysis suggested that the risk of 3‐month unfavorable outcome in RLHI patients complicated with hypoproteinemia during hospitalization was 6.58 times higher than those without hypoproteinemia. Serum albumin, which is the most abundant protein in human blood plasma, is synthesized in the liver. It has many physiological functions, such as regulating colloid osmotic pressure, transporting endogenous and exogenous ligands, anti‐inflammation, anti‐oxidation, and anti‐apoptosis (Boldt, 2010). Experimental studies have demonstrated that albumin therapy had a variety of neuroprotective effects, such as improving neurological function and reducing the volume of cerebral infarction and brain edema in animals with AIS (Belayev et al., 2001, 2005). Observational studies suggested that higher serum albumin levels measured within 36 h after stroke onset were associated with a reduced risk of in‐hospital death and 3‐month unfavorable outcomes (Dziedzic et al., 2004; Vahedi et al., 2011). It is also reported that lower baseline serum albumin levels in AIS patients was associated with a higher risk of hemorrhagic transformation (Che et al., 2017; Wang et al., 2019), and higher incidence of pulmonary infection in observational studies (Dziedzic et al., 2006; Yang et al., 2020). However, the ALIAS (albumin in AIS) part 1 and 2 trials and the combined data analyses showed that intravenous infusion of 25% human serum albumin (2 g/kg) was not associated with improved outcome at 90 days and was associated with the increased incidence of intracerebral hemorrhage and pulmonary edema (Martin et al., 2016). Theoretically, the neuroprotective effects of albumin such as increasing colloidal osmotic pressure to reduce brain edema, as well as anti‐inflammation, anti‐oxidation, and anti‐apoptosis might be beneficial to LHI, but there is still a lack of evidence of clinical trials for the treatment of human serum albumin in patients with LHIs. It is worth noting that RCTs conducted in intensive care unit patients have demonstrated the safety of resuscitation with 20% albumin and its effectiveness in the treatment of patients with severe sepsis or septic shock (Caironi et al., 2014; Mårtensson et al., 2018). Further study is warranted to determine whether 20% albumin is safe and effective in improving the clinical outcome of patients with LHIs. In our study, hypoalbuminemia was defined as serum albumin level <35 g/L during hospitalization. So early nutritional assessment and nutritional support, monitoring of dynamic changes of serum albumin levels, and maintaining serum albumin level ≥35 g/L might be appropriate in improving the clinical outcomes in RLHI patients.

It is worth noting that LHI patients who were current smokers had more favorable outcomes than nonsmokers. A systematic review also found that a history of smoking is one of the protective factors for MBE in patients with AIS (Miao et al., 2020). The protective mechanism of smoking on LHI remains unclear, and it is speculated that nicotine, one of the main components of cigarettes, might have a neuroprotective effect on ischemic brain injury (Pacher & Haskó, 2008). Previous experimental studies have demonstrated that the activation of the endocannabinoid system may play an important role in the neuroprotective effects of nicotine, which increases the release of endocannabinoid and leads to lower body temperature, thereby inhibiting the inflammation and reducing brain edema (Chen et al., 2013; Panikashvili et al., 2001).

### 4.1 Limitations

The results of the present study should be interpreted with caution given its limitations. First, it was a single hospital‐based study, with limited generalizability. Some severe RLHI patients might not be hospitalized, especially those who died before being admitted to the hospital, so we could not exclude inclusion bias in this study. Second, our study only explored acute complications of RLHI patients during hospitalization; we did not include subacute and chronic complications such as dysphagia and the needs for percutaneous endoscopic gastrostomy, depression, and cognitive impairment, because these complications are beyond the scope of the current study. Third, in our stroke unit, some critically ill patients were given up treatment by their relatives and died within a few days after admission, especially those patients who come from rural areas. In addition, most of the survivors with severe disability were taken home directly or opted for hospice care units, the remaining survivors were rapidly transferred to the rehabilitation hospitals or rehabilitation units that were close to their families. As a result, the median length of stay for patients with RLHI is quite short in our study. However, it is reported that neurological complications mainly occur within 48–72 h of stroke onset, whereas medical complications usually develop within the first 1–2 weeks after stroke (Balami et al., 2011; Kumar et al., 2010). As we included RLHI patients admitted within 24 h from symptoms onset with a median length of hospital stay of 14 days, the occurrence of most acute complications could be observed during hospitalization. Fourth, in the present study, hemorrhagic transformation was classified into two types according to the ECASS II criteria. However, it might be more useful to report clinically relevant cerebral hemorrhage and classify the hemorrhagic transformation in more detail according to the SITS‐MOST criteria (Rao et al., 2014). Besides, because of the poor adherence to long‐term follow‐up in RLHI patients, we only conduct a 3‐month follow‐up so that the long‐term effect of the major complications remains unclear. Moreover, we performed the follow‐up by telephone interview or a mailed questionnaire instead of a clinic visit, which may result in reporting bias. Finally, the sample size of our study was relatively small, and the favorable outcome group had only 42 cases. Well‐designed studies with large sample sizes are needed to construct and validate a new prognostic predictive model for RLHI patients.

## 5 CONCLUSIONS

Most of the RLHI patients have at least one stroke‐related complication during hospitalization, and nearly three‐quarters suffered unfavorable outcomes. Only MBE, pulmonary infection, and hypoalbuminemia are independently associated with 3‐month unfavorable outcome. Physicians need to pay more attention to early prevention, identification, and treatment of the major complications to improve the clinical outcomes of patients with RLHI.

## AUTHOR CONTRIBUTIONS

Jie Li and Ping Zhang conceived and designed the study, acquired the funding, collected, analyzed, and interpreted the data, as well as drafted the manuscript. Jie Li, Ping Zhang, Xingrong Luo, and Ju Zhou participated in study administration, investigation, and data collection. Jie Li, Hong Chen, and Yingying Liu contributed to study design, funding acquisition, and study administration. Hong Chen, Yingying Liu, and Chun Wang contributed to study design, administration, and supervision. All authors critically revised the manuscript for important intellectual content and approved the final manuscript.

## CONFLICT OF INTEREST STATEMENT

The authors declare that there is no conflict of interest.

### PEER REVIEW

The peer review history for this article is available at https://publons.com/publon/10.1002/brb3.3095.

## Data Availability

The data and materials that support the findings of this study are available from the corresponding author upon reasonable request.
